# Psilocybin for Depression: From Credibility to Feasibility, What’s Missing?

**DOI:** 10.3390/ph16010068

**Published:** 2022-12-31

**Authors:** Antonio Munafò, Davide Arillotta, Guido Mannaioni, Fabrizio Schifano, Renato Bernardini, Giuseppina Cantarella

**Affiliations:** 1 Department of Biomedical and Biotechnological Sciences, Section of Pharmacology, University of Catania, 95123 Catania, Italy; 2 Department of Neurosciences, Psychology, Drug Research and Child Health, Section of Pharmacology and Toxicology, University of Florence, Viale G. Pieraccini, 6, 50139 Florence, Italy; 3 Psychopharmacology, Drug Misuse & Novel Psychoactive Substances Research Unit, School of Life & Medical Sciences, University of Hertfordshire, Hertfordshire AL10 9EU, UK; 4 Unit of Clinical Toxicology, Policlinico G. Rodolico, School of Medicine, University of Catania, 95123 Catania, Italy

**Keywords:** psilocybin, depression, clinical trials, methodological issues, ethical concerns

## Abstract

Psilocybin has been suggested as a promising transdiagnostic treatment strategy for a wide range of psychiatric disorders. Recent findings showed that psychedelic-assisted/”psycholitic” psychotherapy should provide significant and sustained alleviation of depressive symptoms. However, to date, there have been several study limitations (e.g., small sample sizes, blinding, limited follow-up, highly screened treatment populations) and some health/political issues, including practitioners’ experience, lack of standardized protocols, psychedelics’ legal status, ethical concerns, and potential psychological/psychopathological/medical untoward effects. The focus here is on a range of clinical and methodological issues, also aiming at outlining some possible suggestions. We are confident that newer evidence, more precise protocols, and eventual reclassification policies may allow a better understanding of the real potential of psilocybin as a transdiagnostic therapeutic molecule.

## 1. Introduction

Psychiatric patients’ unmet needs, mental health paucity of resources, as well as psychopharmacology and psychotherapeutic limitations, have led to the investigation of potential novel interventions. Despite decades of relative obscurity, classical psychedelic drugs have been anew investigated in observational, open-label, and randomized controlled clinical studies. Specifically, the classic serotoninergic psychedelics effects have been studied in psychiatric patients and healthy volunteers, with resounding and heartening results [1]. The current wave of psychedelic research has primarily involved psilocybin [2], a molecule that elicited the greatest interest due to its fast-acting properties [3] as well as its apparent safety profile [4] and low potential for abuse [5]. Based on the available preliminary evidence, alongside the current limitations of depression treatment options, the United States Food and Drug Administration granted the “breakthrough therapy” status to psilocybin in 2019, asserting that exploratory data indicate that this compound may provide a meaningful improvement over the present therapies for depression and treatment-resistant depression (TRD).

However, even some supporters of psilocybin worry that the enthusiasm generated might be overstated, given that clinical studies to date have been limited in scope and challenged by several methodological issues, so their data are not and should not be designed to be generalized outside the setting in which they were produced [6]. Therefore, the use of psilocybin in research settings may putatively lead patients to seek it in the community to self-medicate outside of controlled clinical settings and without the guidance of well-trained therapists. Indeed, an escalating increase of retreat centers claiming to provide psilocybin-related levels of emotional breakthrough and spiritual development has recently been observed [7,8,9].

Conversely, several clinical, pharmacological, and methodological challenges relating to these early trials need to be better understood and are discussed here. Particular regard will be given to the therapists’ clinical experience, acute and long-term psilocybin safety issues, and published studies’ methodological limitations.

## 2. How Does It Work? A Summary of the Proposed Mechanisms

Hypotheses concerning the mechanism of action of psilocybin are multifaceted due to the different levels at which the issue can be analyzed. The commonly referenced overarching mechanistic neurobiological principle is that psilocybin acts as an agonist of the 5-Hydroxytryptamine 2A receptor (5-HT2AR), which has been convincingly demonstrated as necessary for psychedelic drug effects [10,11,12], specifically for the propensity to visual hallucinations associated with psilocybin [13]. However, it is not entirely clear which signaling pathways are predictive of the therapeutic effect [14].

The excitatory 5-HT2AR is highly expressed on the cell bodies and apical dendrites of large pyramidal neurons concentrated in the layer V of the cortex with the highest levels in areas crucial for sensory processing, cognition, and mood regulation [15,16]. The activation of 5-HT2ARs leads to a massive depolarization and rapidly repeated firings of these neurons resulting in a profound dysregulation of spontaneous cortical activities and a subsequent state of extreme desynchronization and enhanced entropy [17,18]. In particular, recent neuroimaging and psychopharmacological studies have shown that psilocybin reduces the stability and integrity of well-established brain networks critical for integrating information that serves as a basis for different aspects of complex cognitive functions [17,19,20]. One of these “brain hubs” is known as the default mode network (DMN), a key interconnected set of cortical nodes involved with self-perception and self-awareness, typically hyper-engaged in different psychiatric disorders, whose activity is selectively reduced by psilocybin [21,22,23,24]. Collectively, these studies revealed a dramatic change in global brain connectivity, namely synchronization of sensory networks and a disintegration in associative ones, with a renovated tuning of habits of thoughts and behavior.

Hence, one could argue that there is conceptual evidence that the therapeutic effects of psilocybin are mediated in a different way from those of serotonin-acting antidepressant drugs. The latter provide a “buffer” against stress by strengthening serotonin function at the 5-Hydroxytryptamine 1A receptor (5-HT1AR), whereas psilocybin works by resetting the brain processes underpinning the depressive thinking, leading to enduring therapeutic benefit [25]. To this respect, both preclinical and human studies established that 5-HT1AR ligands can modulate 5-HT2AR-mediated effects, with considerable pharmacological and clinical implications [26,27]. Additionally, different studies suggest that, similarly to ketamine, psilocybin may trigger in layer V pyramidal neurons expressing 5-HT2ARs a “glutamate surge” deemed relevant to its antidepressant effects and instrumental in promoting neurogenesis [28,29].

However, as clinical studies go ahead, researchers should pursue the objective of better understanding the real effects of psilocybin in order to answer cumbersome questions still outstanding, including: How much is just pharmacology? Is psychotherapy really necessary in association with psilocybin? Can the alleged therapeutic properties be dissociated from the subjective effects? [18] How do personality and mood effects in humans back-translate to animals? [30] How important is psilocybin-related mystical experience? Does the content of experience matter? [31,32,33]. The last question of whether the mystical experience is needed is particularly important. It would be intriguing to evaluate psilocybin’s putative effects with the mystical psychedelic experience being blocked or at least attenuated.

## 3. The Clinical Experience to Date

The new process of resuming clinical research with psychedelics was driven by studies assessing the effects of psilocybin-assisted psychotherapy in cancer patients with life-threatening diagnoses and symptoms of depression and/or anxiety. One of these, a randomized, double-blind, crossover trial conducted by Griffiths et al. showed that high-dose of psilocybin (22 or 30 mg/70 kg) produced large and sustained decreases in clinician- and self-rated measures of depressed mood and anxiety [34]. Consistently, a double-blind, placebo-controlled, crossover trial reported by Ross et al. confirmed the robust and enduring anxiolytic and antidepressant effects of psilocybin-assisted psychotherapy in patients with cancer-related psychological distress [35]. Although according to some authors [36], psilocybin users seem to report less intense changes in attitudes towards death compared to other psychedelics (e.g., ayahuasca, DMT). The results of the previously cited end-of-life trials led to open-label and pilot randomized clinical trials of psilocybin in depressed patients.

Indeed, an open-label, single-arm feasibility study assessed the effects of two sessions of psilocybin-assisted psychotherapy, separated by one week, in 12 participants with TRD [37]. Results revealed significant and sustained reductions in depressive symptoms measured by the Quick Inventory of Depressive Symptomatology Self-Report (QIDS-SR) [38]. Robust results were also reported on trait anxiety and anhedonia measures. Though this pilot study provided meaningful insights into examining the treatment safety and feasibility, the results were significantly biased by the absence of a control group, favorable expectancy effects, and participant self-selection biases.

More recently, Davis and colleagues reported findings from a randomized controlled trial (RCT) with a waitlist design assessing the effects of psilocybin-assisted therapy in patients with moderate-to-severe major depressive disorder (MDD) [39]. Across the entire sample, reductions in depression at the QIDS-SR remained clinically significant at the 4-week follow-up. The remission rate, defined as a Hamilton Depression Rating Scale (HAM-D) score < 7, was significant by week 4 [39,40]. Even in this study, there were relevant limitations, for instance, the delayed waitlist design did not control for preparatory and post-session psychotherapy and expectancy effects. In order to improve upon the related research methods, Carhart-Harris et al. conducted a 6-week-phase II double-blind RCT in 59 patients with moderate-to-severe MDD comparing the antidepressant effects of psilocybin-assisted psychotherapy vs. escitalopram, an approved selective serotonin reuptake inhibitor (SSRI) antidepressant [41]. The primary study outcome, a between-group difference in mean changes in QIDS-SR scores from baseline to week 6, did not reach statistical significance. Nevertheless, there were meaningful differences in the rate of response and remission, favoring the psilocybin group over the escitalopram group. Indeed, significant between-group differences in secondary measures of change scores were identified, though the study was under-powered to verify the hypothesis of the superiority of either treatment, and the analyses of secondary outcomes were not corrected for multiple comparisons. The aforementioned study was, however, limited by several important methodological issues. First, the study was not placebo-controlled, hence lacking the ability to verify whether either treatment group would show superiority compared with the placebo. In addition, the duration of escitalopram treatment was considerably shorter than that typically used in clinical practice in view of the delayed antidepressant therapeutic effect [42]. Further, the known participant-selection bias, alongside the expectancy effects not having been controlled, limited the generalizability of results. Very recently, Goodwin et al. carried out a phase 2b double-blind RCT to compare the safety and efficacy of psilocybin at doses of 25 mg or 10 mg with doses of 1 mg in 233 patients with TRD after 2 weeks of wash-out period from their previous antidepressant treatment. The change from baseline at week 3 in the Montgomery–Åsberg Depression Rating Scale (MADRS) total score was significantly better with 25-mg dose than with a 1-mg dose, while no significant differences between the 10-mg dose and the 1-mg dose were detected. Although this trial was designed to address some limitations of previous pilot studies, the main methodological concerns have not been overcome. In addition, along with headache, nausea, fatigue, and dizziness, some participants had suicidal ideation or self-injurious behavior, mainly in the 25-mg and 10-mg groups. The new-onset or worsening of preexisting suicidality with psilocybin reported in this study demands clinical vigilance in future trials [43]. Hence, no definitive conclusions could be drawn about the antidepressant effects of psilocybin due to the study design, the methodological limitations, and both the length of treatment and dosage uncertainties.

## 4. Safety and Inclusion Concerns

Notwithstanding the early results, clinical data provide only a narrow perspective of how effective psilocybin might be as a generalizable treatment for depression since efficacy, safety, and tolerability data of published trials should be appraised in the context of their relevant methodological limitations. In this regard, placebo control and the integrity of blind represent pernicious issues. Indeed, psychedelics are associated with vivid perceptual disturbances and these effects are, of course, only identified in those administered with the active compound. All attempts made to deal with these confounders have indeed failed [44], which could be a reason for concern if psilocybin-assisted psychotherapy will enter real-word practice. No data are available regarding the molecule’s long-term efficacy and effectiveness. These are very relevant clinical and safety concerns that could arise from the escalating number of treated patients. Current studies suggest that psilocybin, used in controlled settings with well-screened participants who are offered appropriate preparation, supervision, and follow-up represents a safe, well-tolerated, and effective treatment for depressed patients and unlikely causes sustained psychiatric complications or serious adverse events. However, individuals with psychiatric comorbidities have been selectively excluded. On the other hand, newer findings suggest that inclusion criteria, in certain circumstances, could even be expanded [45]. Likewise, attempts or suicides attributed to psilocybin have only recently been reported [43], with participants with active suicidal ideation or medically serious suicide attempts having been explicitly excluded [46]. Moreover, another sensible research gap is related to the exclusion from previous studies of both adolescent and drug-misusing patients [47].

Additionally, patients with significant underlying cardiovascular, neurological, liver or kidney comorbidities have largely been excluded and it is unclear whether psilocybin’s safety profile may be impacted by underlying medical disorders or by drug–drug interactions. In this respect, preliminary evidence suggested that the subjective effects of psychedelics could be attenuated by ongoing treatment with SSRIs. Therefore, it has become necessary to discontinue the medication with the risk of worsening the underlying depression and/or inducing withdrawal or discontinuation reactions [48,49]. To date, there has been only one controlled study combining psilocybin with an SSRI. Specifically, Becker et al. conducted a double-blind, placebo-controlled, crossover-design study to investigate the response to psilocybin (25 mg) in healthy subjects after pretreatment for two weeks with escitalopram or placebo. Contrary to previous data, escitalopram pretreatment did not significantly attenuate the acute positive mood effects of psilocybin and reduced its related adverse effects [50]. These results need to be confirmed in follow-up clinical trials with a longer antidepressant pretreatment time with the aim of elucidating the interactive effects on therapeutic outcomes and whether antidepressant treatment should be maintained or stopped before psilocybin administration. A similar concern applies to patients who are already on 5-HT2AR-blocking antipsychotic drugs such as quetiapine and olanzapine [51,52,53].

The above-mentioned selection criteria are justifiable in terms of optimizing safety. Nevertheless, their exclusiveness might not reflect appropriately the heterogeneity of the population that could benefit from this putative novel therapeutic intervention. As a matter of fact, for most psilocybin studies, the population could be described, in broad terms, as “mainly Caucasian, with a high degree of education, living in major urban centers”, in sharp contrast with the reality of MDD [54]. As a developing field, the psychedelic research community should comply with the ethical imperative to conduct more inclusive and equitable studies.

## 5. Challenges, Limitations, and Suggestions

Despite the recent widespread media coverage around the psychedelic renaissance, a renaissance characterized by an unreserved promotion of the potential benefits and applications of psilocybin as a therapeutic option, little has been written about the challenges this field faces when put under a critical and methodological microscope [55]. In fact, psychedelic trials are particularly challenging as they have to address methodological issues inherent to both psychotherapeutic and clinical pharmacological research, as well as limitations related to expectancy effects and effective condition masking [44]. 

Psychedelic researchers should always pay attention to their attempts to mitigate expectancy, defined as an observable neurobiological effect that may be responsible, at least in part, for the clinical improvements observed [56]. Across clinical research contexts, expectancy could significantly affect clinical outcomes. Specifically, expectations can be split into process expectations (e.g., relating to any acute drug effect during experimental intervention) and outcome expectations, referring to whether treatment is anticipated to reduce symptoms [57]. These aspects are particularly pertinent in psilocybin-assisted psychotherapy trials, whose fundamental elements contribute to changes in participants’ process and outcome expectations throughout the entire process [58]. 

In such conditions of high expectancy, supported by amplified external media sources, positive expectations likely lead to emphasized treatment effects in the active arm [59]. Similarly, the latter may produce detrimental effects when disappointed patients gain knowledge of being allocated in the control arm [60]. In other words, negative outcome expectations, due to the awareness of assignment to a treatment that patients believe is unlikely to improve symptoms, can worsen clinical outcomes [61,62]. This is particularly salient for high-dose psilocybin trials, in which subjective symptoms are especially pronounced and relatively easy to discern [63]. To this respect, it would be appropriate to exclude participants with a previous history of use with psychedelics [64,65]. Notably, expectations, both of process and outcome, have been rarely evaluated and often thought of as a nuisance rather than an important ingredient of the therapeutic process with a consequent overestimation of treatment effects [66]. Hence, investigators should manage expectations whilst emphasizing the uncertainty regarding the treatment efficacy and measuring participants’ treatment expectations through established measures, such as the Stanford Expectations of Treatment scale [67], both at the baseline and after drug administration. Collectively, to date, the interrelated methodological challenges regarding blinding and expectancy effect have raised critical limitations to the interpretability of the research [68].

Moreover, the structure of psilocybin-assisted psychotherapy involves at least three phases: Preparation session, single or multiple drug dosing session, and integration session after drug administration [69]. Although all participants putatively receive the same type and frequency of psychological therapy, one could argue that the content is possible to change based on treatment allocation. In fact, some of the administered questionnaires refer to those rating experiences that are usually considered unique to psilocybin, for instance the Mystical Experience Questionnaire (MEQ) [70] may indeed modify the participant’s belief about treatment allocation (e.g., inducing unintentional respondent’s reaction biases) [71]. Furthermore, it is not difficult to foresee a situation where the therapist is aware of whether the patient has been allocated to the active arm or not, delivering differential therapy across treatment groups [72]. To this respect, are the “integration” psychotherapeutic sessions appropriate for the control group as well? Additionally, is the therapeutic alliance between patient and practitioner to be considered a mediator or a confounder? The first data on the relative necessity of psychotherapeutic integration after a psychedelic session have recently been published [73]. Although concerning ayahuasca, which contains multi-target psychoactive compounds, these preliminary findings may lead some to reconsider/downsize the actual impact of this kind of integrated approach. In this context, future trials should compare the actual mood-enhancing effects of psilocybin alone and as an adjunct to psychotherapy. Moreover, while it is true that therapists’ positive attitudes and beliefs about psilocybin have been associated with greater openness to involving patients with psilocybin-assisted psychotherapy [74], potential limitations in terms of accessibility to care can be anticipated. For example, as noted by some authors, economic and other treatment engagement issues can act as barriers to receiving psychotherapy assistance, particularly in poorer populations and minority groups [75,76,77,78].

In psychedelic clinical trials, experimental challenges related to expectancy and placebo/nocebo effects primarily derive from unsuccessful blinding [79,80]. Therefore, different methodological procedures designed to decrease the participant subject’s confidence in guessing his/her assigned treatment arm should be implemented. Furthermore, although previous studies provided two psilocybin drug sessions, one could wonder if multiple treatments administered within a short period of time may produce more significant or sustained therapeutic effects compared to a single/only a few psychotherapeutic sessions. Studies with a single dosing session are likely to be superior in maintaining successful blinding [81]. Moreover, a stronger study design should include, apart from an inactive control, an active control condition, such as an effectively psychoactive placebo able to simulate the subjective and acute effects of psilocybin [58]. Theoretically, an active placebo that mimics psychedelic effects without providing therapeutic benefit might appear appropriate for this purpose, although one would argue that it may well be the “mystical state” itself that may drive the beneficial effects [31,32,82]. Finally, some subjects are inclined towards a rational philosophical approach that strongly denies even the mere existence of a “mystical experience”, including its role in the healing process [83,84,85,86]. Hence, one could hope that future therapeutic options will consider the possibility of assessing the effectiveness of non-hallucinogenic but still psycho-plastogenic substances [87]. To date, however, those approaches aimed at specifically addressing this methodological challenge have proven unsuccessful [35,88,89]. The use of low-dose psychedelics as part of a potential active control condition represents a promising starting point to improve participant masking and balance treatment expectations among conditions [34,41]. This approach could be assisted by an incomplete disclosure of certain aspects of the study design, namely a neutral explanation of drug effects, hence, enhancing masking success [90,91].

## 6. Final Concerns

According to some [1], the resurrection of psychedelic compounds represents one of the most important initiatives in psychiatry in recent decades. Psychiatrists and patients are excited about the alluring promise of a “resolutive” treatment that can achieve a meaningful response through a short-term integrated clinical protocol.

However, a range of methodological, clinical, and safety concerns have been highlighted here, and these concerns may well temper the levels of excessive enthusiasm associated with the recent psychedelic renaissance. Further issues of concern relate as well to the risks of psychopathological consequences associated with a single/multiple intakes of psychedelics (e.g., self-harm, paranoid disturbances, long-term depersonalization/derealization, excessive mood enhancement, persisting hallucinogen use disorders) [92,93]. Therefore, considering the wide and, in some cases, unpredictable effects that this approach may have, rigorous and shared ethical and practical standards could be needed to ensure its safe and responsible clinical use [94,95].

Moreover, we think that the importance of psychedelics’ dosing in terms of efficacy and safety should be better discussed. The perception of psychedelic intake relative safety is here regarded as misleading. This could take the general public, but especially so vulnerable clients, to conclude that it may be “ok” to self-administer with a dose, or maybe a “microdose” [96] of psychedelics, either for mood control or cognitive performance improvement purposes [97,98,99]. Finally, there are clear concerns relating to the toxicological drug test screening (with psychedelics not typically being identified [100]) in cases of potential abuse/misuse. Overall, it clearly appears that the future of psychopharmacological research should focus on safe practices and inclusive public policies [101,102] without an exasperated emphasis on spirituality.

## Data Availability

Not applicable.
